# Optical imaging of the peri-tumoral inflammatory response in breast cancer

**DOI:** 10.1186/1479-5876-7-94

**Published:** 2009-11-11

**Authors:** Akhilesh K Sista, Robert J Knebel, Sidhartha Tavri, Magnus Johansson, David G DeNardo, Sophie E Boddington, Sirish A Kishore, Celina Ansari, Verena Reinhart, Fergus V Coakley, Lisa M Coussens, Heike E Daldrup-Link

**Affiliations:** 1 Department of Radiology and Biomedical Engineering, University of California, San Francisco, USA; 2 Department of Pathology and Cancer Research Institute, University of California, San Francisco, USA

## Abstract

**Purpose:**

Peri-tumoral inflammation is a common tumor response that plays a central role in tumor invasion and metastasis, and inflammatory cell recruitment is essential to this process. The purpose of this study was to determine whether injected fluorescently-labeled monocytes accumulate within murine breast tumors and are visible with optical imaging.

**Materials and methods:**

Murine monocytes were labeled with the fluorescent dye DiD and subsequently injected intravenously into 6 transgenic MMTV-PymT tumor-bearing mice and 6 FVB/n control mice without tumors. Optical imaging (OI) was performed before and after cell injection. Ratios of post-injection to pre-injection fluorescent signal intensity of the tumors (MMTV-PymT mice) and mammary tissue (FVB/n controls) were calculated and statistically compared.

**Results:**

MMTV-PymT breast tumors had an average post/pre signal intensity ratio of 1.8+/- 0.2 (range 1.1-2.7). Control mammary tissue had an average post/pre signal intensity ratio of 1.1 +/- 0.1 (range, 0.4 to 1.4). The p-value for the difference between the ratios was less than 0.05. Confocal fluorescence microscopy confirmed the presence of DiD-labeled cells within the breast tumors.

**Conclusion:**

Murine monocytes accumulate at the site of breast cancer development in this transgenic model, providing evidence that peri-tumoral inflammatory cell recruitment can be evaluated non-invasively using optical imaging.

## Background

The intimate association between cancer and inflammation was first identified over a century ago. The role of the immune system in modulating carcinogenesis is complex; some aspects of the immune response are protective, while others are pro-tumorigenic. Several findings support the suggestion that inflammation plays a role in promoting breast cancer. From an epidemiologic perspective, immunocompromised individuals, such as organ transplant recipients, have a lower incidence of breast cancer [1,2]. It has also been noted that as breast cancer progresses, there is a corresponding increase in the number of leukocytes, both of lymphoid and myeloid origin, surrounding the tumor [3].

There are several proposed mechanisms by which the immune response may promote breast cancer development. Infiltrating immune cells elaborate cytokines, chemokines, metalloserine and metallocysteine proteases, reactive oxygen species, and histamine, all of which augment tumor remodeling and angiogenesis [4-6]. Chronic B-cell activation and helper T-cell polarity towards the Th2 subtype are also thought to play roles in supporting tumorigenesis [7-10].

Tumor associated macrophages/monocytes are also thought to promote tumor development through the elaboration of tumor growth factors, proangiogenic substances, matrix degrading proteins, and DNA-disrupting reactive oxygen species [11-15]. In the mouse mammary tumor virus - polyomavirus middle T antigen (MMTV-PymT) transgenic mouse model, macrophage infiltration into premalignant breast lesions is associated with tumor progression [16]. Moreover, limiting macrophage infiltration reduces tumor invasion and metastasis in this model [17]. In humans, elevated levels of CSF-1 and exuberant macrophage recruitment are associated with poor prognosis [13,15,18].

The MMTV-PymT transgenic murine model of breast cancer is a well characterized model which recapitulates human disease, with progression from hyperplasia to invasive carcinoma and metastatic disease at ~115 days of life [3,18]. As described above, a significant inflammatory response, populated by B and T lymphocytes, macrophages/monocytes, and mast cells, accompanies breast tumor development.

With this background, the purpose of this study was to use optical imaging to non-invasively monitor the peri-tumoral inflammatory response in the MMTV-PymT transgenic mouse by tracking monocyte recruitment. A technique based on the detection of fluorescence, optical imaging (OI) is a relatively new modality in the clinical setting. Compared with other imaging modalities, optical imaging is inexpensive, easy and fast to perform, highly sensitive, and radiation-free. In addition, breast cancer patients have been previously scanned using optical imaging; initial results indicate that this technique may supplement mammography and magnetic resonance imaging in breast cancer detection [19,20]. Our group and others have established optical imaging-based "leukocyte scans" by labeling leukocytes with fluorochromes ex vivo, intravenously injecting them into experimental animals, and subsequently tracking the labeled cells with optical technology. These scans have been used to detect and monitor treatment of arthritis [21] and to track cytotoxic lymphocytes to implanted tumors [22].

Optically tracking monocytes to breast tumors in the MMTV-PymT model has several potential utilities. First, the temporal relationship between breast tumor development and inflammation could be better characterized, without having to sacrifice animals. Second, evaluating the extent of monocyte recruitment may have prognostic implications, as described previously. Third the effect of anti-inflammatory and chemotherapeutic regimens on peri-tumoral inflammation and monocyte recruitment could be assessed.

## Materials and methods

### Monocytes

Murine monocytes were obtained from the continuously growing leukemic cell line, 416B (Cell Culture Facility, University of California, San Francisco, ECACC equivalent 85061103) and cultured in Dulbecco's Modified Eagle Medium (DMEM) high glucose medium supplemented with 10% fetal bovine serum and 1% Penicillin/Streptomycin. 416B monocytes were grown in this medium as a non-adherent suspension culture at 37°C in a humidified 5% CO_2 _atmosphere.

### In vitro cell labeling

Triplicate samples of 1, 2, and 4 million monocytes/mL of serum-free DMEM were incubated with a solution of the fluorochrome DiD at a ratio of 5 μL DiD/1 mL DMEM for 15 minutes at 37 degrees C. DiD (C_67_H_103_CIN_2_O_3_S,: Vybrant cell labeling solution, Invitrogen) is a non-targeted, lipophilic, carbocyanine fluorochrome with a molecular weight of 1052.08DA and excitation and emission maximum of 644 nm and 665 nm respectively. The labeled cells were washed 3 times with phosphate-buffered saline (PBS) (pH 7.4) by sedimentation (5 min, 400 rcf, 25°C). The labeled monocytes were placed in the Xenogen IVIS 50 optical imager (Xenogen Corporation, Alameda, CA) and scanned. Flow cytometry using Cytomics FC500 flow cytometer (Beckman-Coulter Inc., Fullerton, CA) was performed on labeled cells to confirm integration of DiD. Triplicate samples of 2 million cells labeled with 5 microliters of DiD were optically imaged at 24 hours to determine persistence of labeling.

### Cell Viability

2 million 416B monocytes in 2 mL DMEM were incubated for 15 minutes with 0-20 microliters of DiD, with the total volume of 20 microliters being completed with ethanol. Trypan blue testing of the labeled cells was then performed to determine viability. Additionally, 2 million 416B monocytes in 2 mL DMEM were incubated for 15 minutes with 5 microliters of DiD, and viability of cells was assessed 24 hours after labeling with trypan blue staining.

### Ex vivo cell labeling

Samples of 10^7 ^monocytes were incubated for 15 minutes with 25 μl of DiD in 5 ml (Concentration: 5 microliters DiD/1 ml DMEM) of serum free DMEM and then washed 3 times with phosphate-buffered saline (PBS) (pH 7.4) by sedimentation (5 min, 400 rcf, 25°C) prior to intravenous injection.

### Animal studies

This study was approved by the animal care and use committee at our institution. All imaging procedures as well as monocyte injections were performed under general anesthesia with 1.5-2% isoflurane in oxygen, administered via face mask. Studies were carried out in twelve mice: six MMTV-PymT trangenic mice (age range 95-115 days) and six FVB/n control mice. For cell injections, either an internal jugular or femoral vein direct cannulation was performed with a 30-guage needle. Labeled cells were suspended in a total volume of 350 microliters of PBS prior to injection. The cell-free DiD infusion was performed by injecting a solution consisting of 5 microliters of DiD and 345 microliters of PBS intravenously. Peripheral blood for flow cytometry analysis was obtained via cardiac puncture.

### Optical Imaging

All optical imaging studies were performed using the IVIS 50 small animal scanner (Xenogen, Alemeda, CA) and Cy5.5 (excitation: 615-665 nm and emission: 695-770 nm passbands) filter set. For in vitro studies, cell samples were placed in a non-fluorescing container. For in vivo studies, mice were anesthetized with isofluorane and placed in the light-tight heated (37 degrees celsius) chamber. After being shaved, the animals were imaged in three positions at all time points: (1) anterior (facing the CCD camera), (2) left lateral decubitus, and (3) right lateral decubitus. Identical illumination parameters (exposure time = 2 seconds, lamp level = high, filters = Cy5.5 and Cy5.5 bkg, f/stop = 2, field of view = 12, binning = 4) were selected for each acquisition. Gray scale reference images were also obtained under low-level illumination. Optical imaging scans were obtained before and at 1, 2, 6, and 24 hours after intravenous monocyte injection. After completion of the scans, the animals were sacrificed via a combination of cardiac puncture and cervical dislocation while under anesthesia. Tissues were immediately harvested for sectioning and microscopic analysis.

### Data analysis

OI Images were analyzed using Living Image 2.5 software (Xenogen, Alameda, Ca) integrated with Igorpro (Wavemetrics, Lake Oswego, OR, USA). Images were measured in units of average efficiency (fluorescent images are normalized by a stored reference image of the excitation light intensity and thus images are unitless) and corrected for background signal. For in vitro image analysis, regions-of-interest (ROI) were defined as the circular area of the tube. For in vivo image analysis, ROIs were placed around breast tumors (MMTV-PymT mice) and mammary tissue (FVB/n controls). The post to pre-injection fluorescence signal intensity (SI post/pre) was then calculated for each ROI.

### Statistical Analysis

All in vitro experiments were performed in triplicates. Data were displayed as means plus/minus the standard error of the mean (SEM). Student t-tests were used to detect significant differences between labeled and unlabeled monocytes (in vitro data) and breast tumor and control mammary tissue (in vivo data). Statistical significance was assigned for p values < 0.05.

### Immune Fluorescence and Confocal Analysis

Tumors were explanted 24 hrs after monocyte injection and preserved in OCT at -80°C. 5 μm thick slides were prepared which were then processed for immunostaining. CD45 immunostaining (eBioscience, San Diego, CA) was performed to visualize murine monocytes in the tumor, while the tumor nuclei were mounted with a mounting medium containing DAPI (Vectashield Mounting medium with DAPI, Vector Laboratories, Burlingame, CA). Confocal analysis was performed using a Zeiss LSM510 confocal microscopy system equipped with krypton-argon (488, 568 and 633 nm) and ultraviolet (365 nm) lasers; images were acquired using LSM version 5. Images are magnified to 10×. The images presented are representative of four independent experiments. All images were converted to TIFF format and arranged using Adobe Photoshop CS2.

### Flow Cytometry

DiD labeled and unlabeled murine monocytes were resuspended in PBS/BSA and incubated for 10 min at 4°C with rat anti-mouse CD16/CD32 mAb (BD Biosciences, San Diego, CA) at a 1:100 dilution in FACS buffer to prevent nonspecific antibody binding. After incubation and washing, the cells were incubated with anti-CD45-PE (pan-leukocyte marker), anti-CD11b-PE (monocyte and macrophage marker), anti-Gr1-FITC (granulocyte marker), and anti-F4/80-FITC (macrophage marker) (eBioscience) for 20 min with 50 μl of 1:100 dilution of primary antibody followed by two washes with PBS/BSA. 7-AAD (BD Biosciences) was added (1:10) to discriminate between viable and dead cells. Data acquisition and analysis were performed on a FACSCalibur using CellQuestPro software (BD Biosciences). DiD was visualized using the FL4 channel.

## Results

### In vitro optical imaging

**Figure 1:**
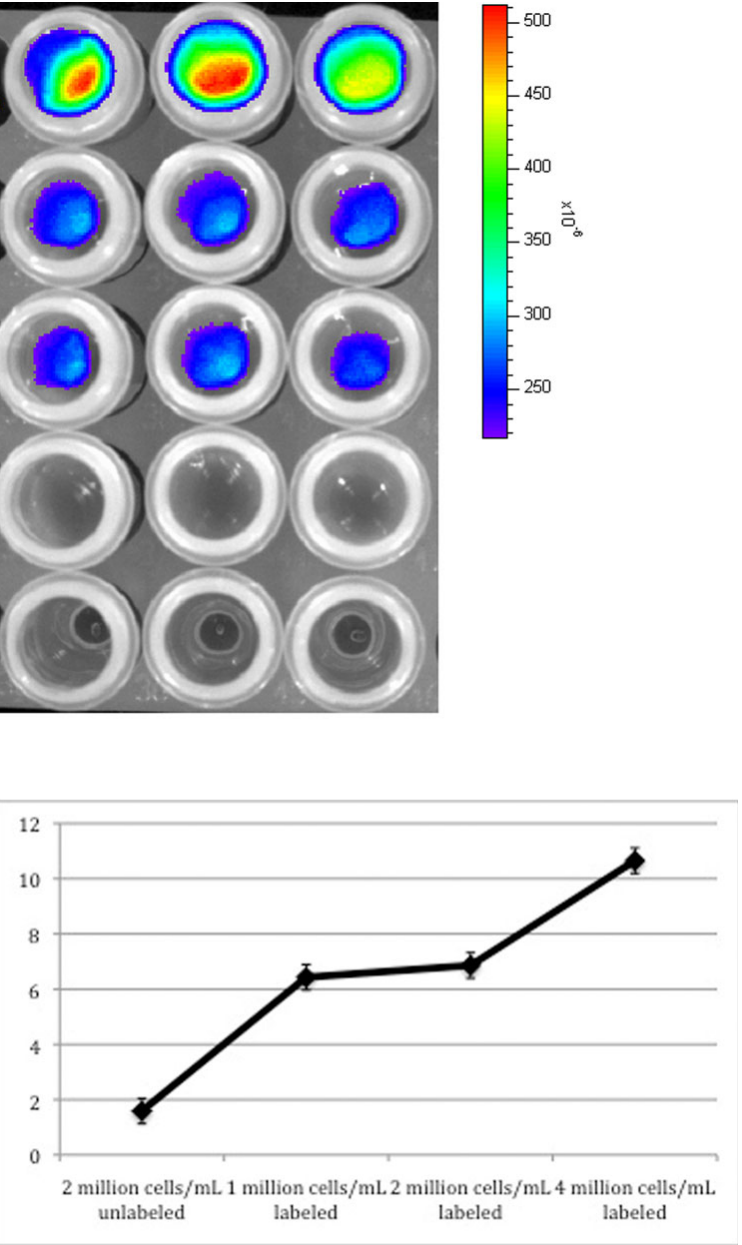
**(a) Optical imaging of DiD-labeled cells immediately after labeling**. First 3 rows: triplicately labeled cells. (Top row: 4 million/cells mL; Second row: 2 million cells/mL; Third row: 1 million cells/mL). Fourth row: unlabeled cells (2 million cells/mL). Fifth row: DMEM alone. (b) Ratio of fluorescence of cells to media (Y-Axis) for each sample of cells (X-Axis). The ratio of labeled cells to media was significantly higher at all concentrations than the ratio of unlabeled cells to media (p < 0.01). Error bars represent standard error of the mean.

OI of DiD-labeled cells at all concentrations demonstrated significantly higher fluorescence from labeled cells compared to that from non-labeled controls (p < 0.01). There was increasing fluorescence from DiD-labeled cells with increasing cell concentration, indicating no quenching effects within the range of evaluated cell concentrations; however, graphically, the increase in fluorescence with cell concentration labeled was not unequivocally linear (Figure 1b). There was no change in the fluorescence of cells imaged at 24 hours compared with those imaged immediately after labeling. Viability of the cells post labeling is shown in Table 1. Cell viability decreased as DiD dose was increased. Trypan blue staining demonstrated 80% viability 24 hours post labeling.

**Table 1:** Cell viability as a function of DiD concentration.

Amount of DiD added	Cell Viability (%)
20 microliters	73

10 microliters	78

5 microliters	82

2.5 microliters	84

1.25 microliters	83

0 microliters	84

Cells alone	90

### Flow cytometry

**Figure 2:**
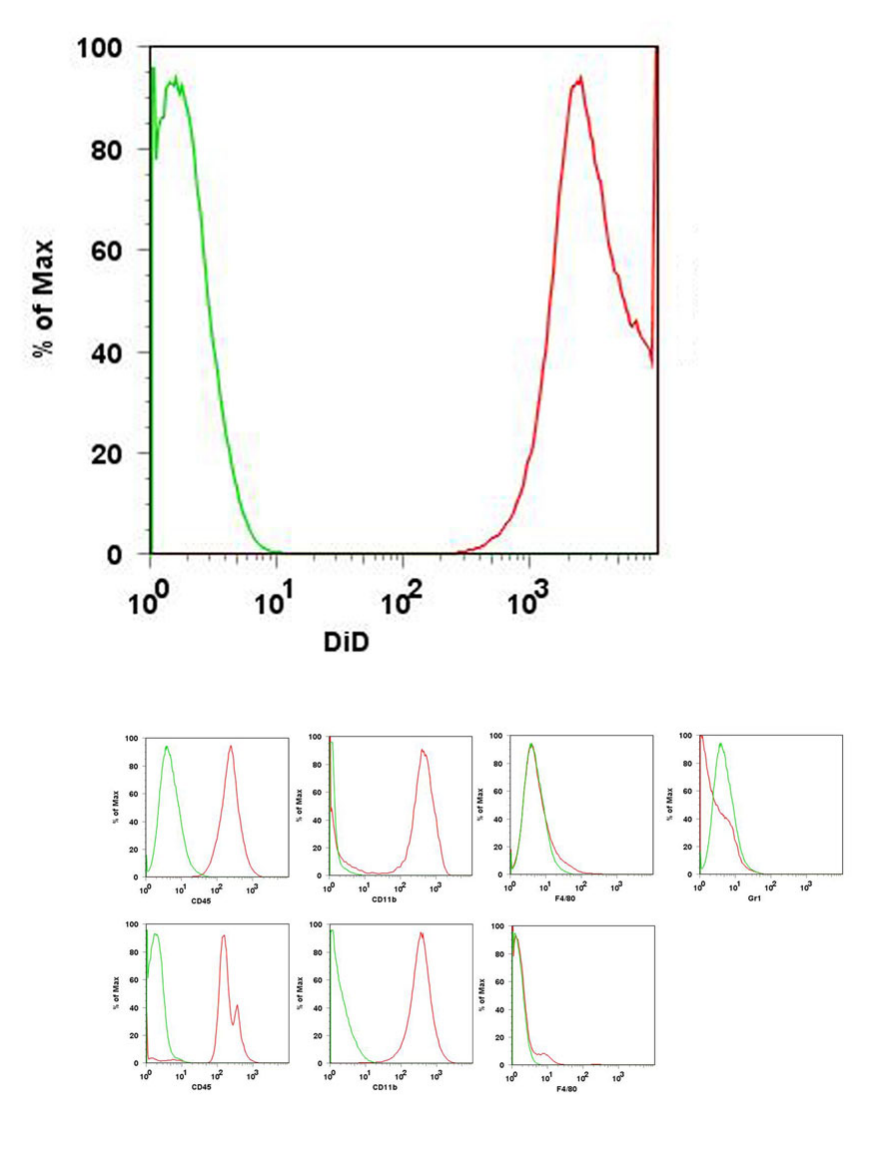
**(a) Flow cytometry for DiD-labeled 416B murine monocytes**. Left peak (green): unlabeled cells, right peak (red): DiD-labeled cells. (b) Flow cytometry characterization of 416B murine monocyte cell line. Top row: 416B cell line, bottom row: peripheral blood monocytes from FVB/n control mice. For all images, the green peak represents unlabeled cells, and the red peak represents labeled cells.

Flow cytometry demonstrated that the monocytes incubated with DiD fluoresced distinctly from unlabeled cells in the fluorescent range of DiD. (Figure 2a) Additional flow cytometry data demonstrated that the monocyte cell line has the same markers as monocytes isolated from peripheral blood; specifically, it is CD45 and CD11b positive and F4/80 negative. The absence of Gr1 fluorescence confirmed that the cell line did not differentiate along the granulocytic pathway. (Figure 2b)

### In vivo optical imaging

**Figure 3:**
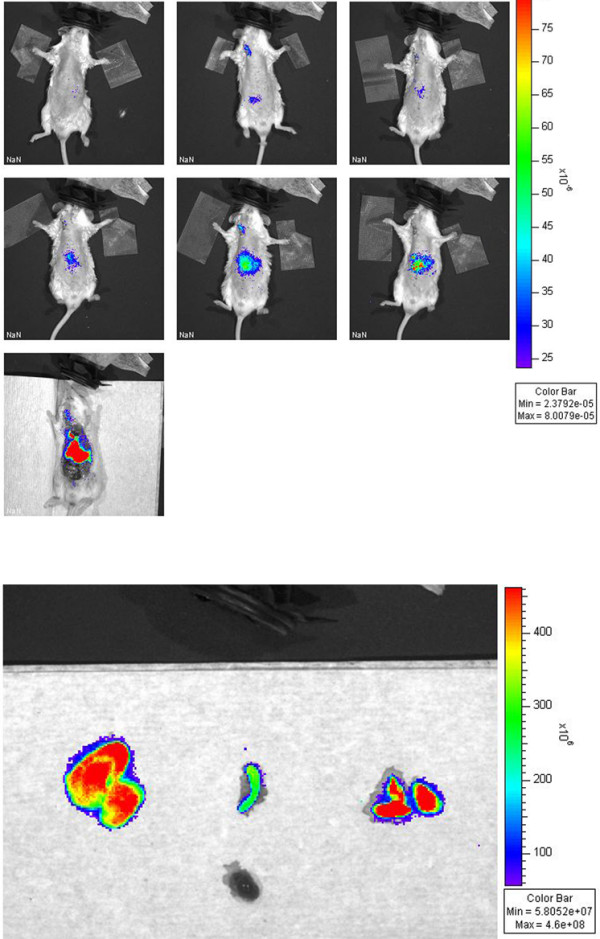
**(a) In vivo optical imaging of a control FVB/n mouse after intravenous injection of DiD-labeled monocytes**. Top row, left to right: pre-injection, 1 hour, and 2 hours post-injection. Middle row, left to right: 6 hours, 12, and 24 hours post injection. Bottom image: post-mortem dissection. (b) Removed organs 24 hours post injection. Left to right: Liver, spleen, lungs, heart. Images are representative of the FVB/n control mice injected with DiD-labeled monocytes.

**Figure 4:**
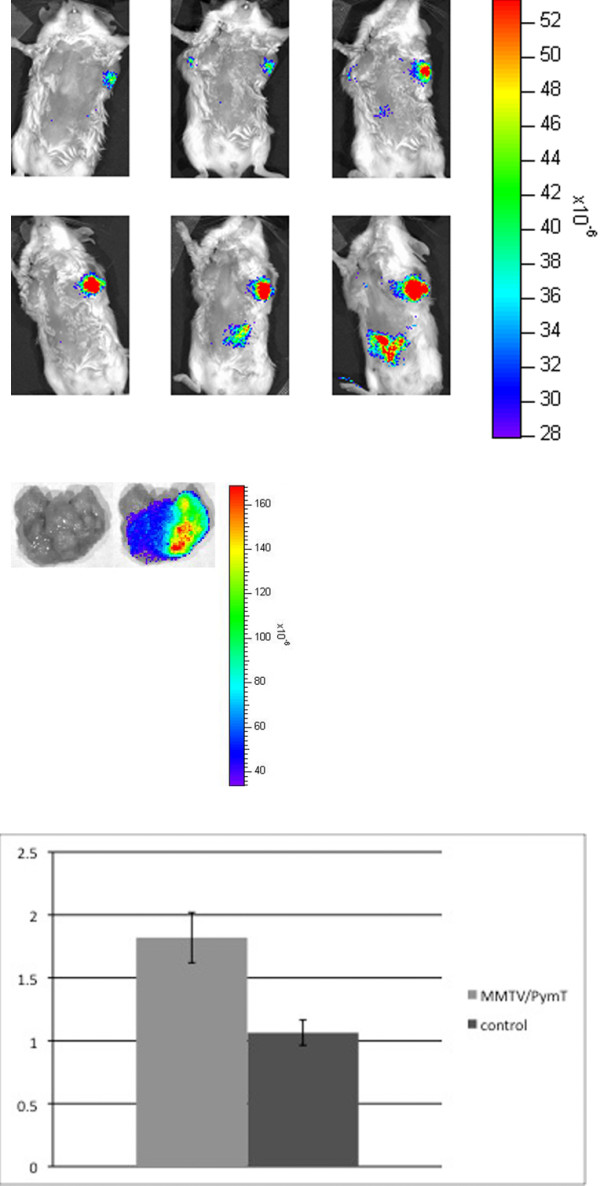
**(a) In vivo optical imaging of a MMTV-PymT mouse after intravenous injection of DiD-labeled monocytes**. Top row, left to right: pre-injection, 1 hour, 2 hours post-injection. Bottom row, left to right: 6 hours, 12 hours, 24 hours post-injection. (b) Optical imaging of explanted left axillary tumor from the same mouse. Left to right: photograph only, fluorescence image. Images are representative of the MMTV-PymT mice injected with DiD-labeled monocytes. (c) Quantitative analysis of fluorescence from breast tumors following injection of DiD-labeled monocytes. The left bar represents the average SI post/pre fluorescence ratio within breast tumors from MMTV-PymT mice, while the right bar represents the average SI post/pre fluorescence ratio within mammary tissue from FVB/n controls. Y-axis: average SI post/pre fluorescence ratio. Error bars represent the standard error of the mean. The difference between the two ratios was statistically significant, with a p-value less than 0.05.

After injecting DiD-labeled monocytes into FVB/n controls, progressively increasing fluorescence was noted in the liver, spleen, and lungs over 24 hours. (Figure 3). The same pattern was observed in MMTV-PymT mice. In addition, MMTV-PymT mice demonstrated increasing fluorescence within tumors over the course of 24 hours (Figure 4). This data is shown quantitatively in figure 4c, which demonstrates an average SI post/pre ratio of 1.8 +/- 0.2 (SEM) in MMTV-PymT breast tumors, with a range of 1.1 to 2.6. Mammary tissue of FVB/n controls had an SI post/pre ratio of 1.1 +/- 0.1 (SEM). The difference between these averages was found to be statistically significant, with a p-value less than 0.05. Injection of free DiD resulted in no increase in fluorescent intensity within the tumor at any time point post-infusion.

### Fluorescence microscopy

**Figure 5:**
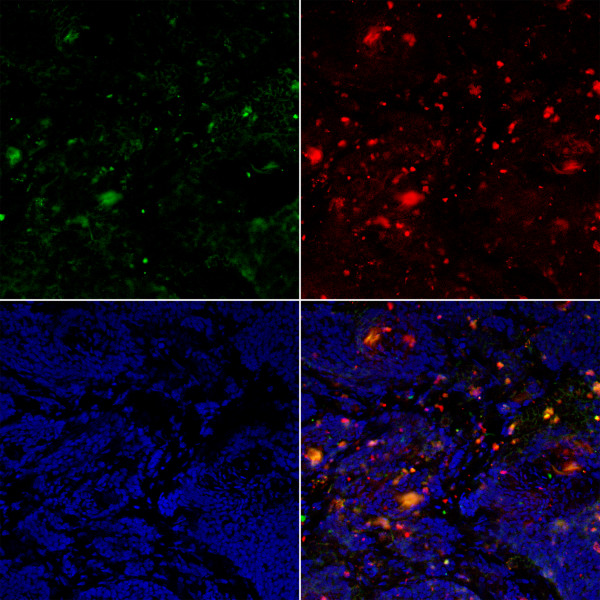
**Immunofluorescence/confocal microscopy**. Top row, left to right: CD45, DiD. Bottom row: DAPI, merged image. Confocal images are representative of the MMTV-PymT control mice injected with DiD-labeled monocytes. Images are at 10× magnification.

Harvested tumors from MMTV-PymT mice were sectioned for fluorescence microscopy. Figure 5 demonstrates cells that fluorescently stain for both CD45 and DiD, thus confirming that injected DiD-labeled monocytes are present within breast tumors. CD45 and DiD signal colocalization, while present in all tumor tissues, was not uniformly distributed across all areas of the tumor specimens observed. Additionally, there were some areas with CD45 positive signal without DiD signal.

## Discussion

The above results demonstrate that after intravenous injection of fluorochrome-labeled monocytes, there was progressive fluorescence within the breast tumors of MMTV-PymT mice, a phenomenon not seen in the mammary tissue of FVB/n control mice. Fluorescence microscopy confirmed that DiD-labeled monocytes were present within breast tumors, though the lack of uniform DiD-fluorescence distribution in the tumor specimens is likely a reflection of the heterogenous distribution of tumor-associated macrophage recruitment within the tumor microenvironment. The scattered presence of CD45 positive but DiD negative regions may either be reflective of endogenous murine monocytes recruited to the tumor simultaneously, or, alternatively, exogenous monocytes that were ineffectively labeled with DiD before intravenous injection. Nonetheless, taken together, it can be concluded that intravenously injected, fluorescently-labeled monocytes accumulate within breast tumors in this transgenic murine model of breast cancer, where they can be visualized with optical imaging technology. Flow cytometry validated the murine monocyte cell line 416B as being a legitimate and relevant cell line for this study, as these cells have expression patterns similar to monocytes isolated from the peripheral blood of control mice.

Thus far, molecular imaging techniques have focused on imaging cancer cells themselves, proteins that are overexpressed by cancer cells, angiogenic markers, or the extracellular matrix surrounding cancer [23-25]. The inflammatory component of cancer biology, on the other hand, has not been a major target of molecular imaging technologies. Inflammation has been evaluated in other contexts, such as in a mouse model of type 1 diabetes [26], and a rat model of arthritis [21]. Inflammatory macrophages in atherosclerotic plaques have also been imaged with magnetic resonance using superparamagentic iron oxide particles [27]. Genetically engineered T lymphocytes have been tracked to animal tumors using microPET technology [28,29]. Superparamagnetic iron-oxide labeling and subsequent MR imaging of immune cells have been employed as a strategy to monitor anti-cancer cellular therapy [30]. Monocytes have been labeled with MR contrast agents and tracked to rat gliomas [31]. However, to our knowledge, this is the first demonstration of tracking fluorescently labeled monocytes to breast cancer using optical imaging.

The mechanism by which these cells are recruited to breast tumors in MMTV-PymT mice is multifactorial, and may be related to vascular permeability and local factors released by tumor cells, stromal cells, and inflammatory cells. Elaboration by these cells of the inflammatory chemokine CCL2 (MCP-1) is associated with both monocyte recruitment and poor prognosis [32,33]. Jin et al. demonstrated a role for integrin alpha 4 beta 1 in the homing of monocytes to tumors. Specifically, the group noted that blocking this integrin in a mouse model of implanted lung cancer suppressed the number of macrophages within tumors and also stunted tumor growth [34]. CSF-1 release by tumor cells is also thought to play a role [35,36]. The relative contributions of these various factors to monocyte recruitment may potentially be further characterized using the imaging technique described here.

There are several limitations to the current study. As this was a proof of principle study, a limited number of animals was used to obtain statistical significance. A larger sample size would provide further characterization of the inflammatory response and monocyte recruitment. Second, while the pathogenesis of breast cancer seen in this animal model closely resembles that in humans, there may be significant differences between the two species. Third, while this technique has potential clinical applications, DiD has not received FDA approval. Given that other cyanine dyes have significant toxicity, further studies will be required to determine the safety of DiD. It should be noted, however, that another cyanine fluorescent dye, Indocyanine Green (ICG), has received FDA approval.

In conclusion, tracking monocytes non-invasively will lead to a better temporal and pathophysiological understanding of the in vivo inflammatory response around breast cancers. Moreover, this imaging technique could be used as a supplemental prognostic tool, given the aforementioned inverse correlation between the degree of monocyte recruitment and prognosis. In addition, the presented technique could streamline the development of novel chemotherapeutic and anti-inflammatory pharmaceuticals for breast cancer treatment [37]. For example, following intravenous injection of fluorophore labeled leukocytes, the efficacy of such agents could be assessed by the degree of monocyte accumulation within tumors. Given the recent development of handheld OI scanners and dedicated OI breast scanners, the imaging technique described here has the potential to directly impact clinical decision making and drug development in the breast cancer arena.

## Competing interests

The authors declare that they have no competing interests.

## Authors' contributions

AKS conducted or took part in all the experiments and was the primary writer of the manuscript. RJK was involved in the in vivo data gathering. ST performed or participated in both the in vitro and in vivo studies. MJ performed the immunofluorescence and flow cytometry studies. DGD conducted the immunofluorescence and confocal microscopy experiments. SEB performed several of the in vitro experiments. SAK was involved in data analysis and wrote part of the manuscript. CA and VR gathered a portion of the in vitro data. FVC was the primary investigator on the T32 training grant and edited the manuscript. LMC was the primary investigator on the NIH grants and Department of Defense grant listed in the acknowledgements section and was involved in the study design. HED-L was involved in the conception of the study and was the primary investigator on Award Number R21CA129725 listed in the acknowledgements section. All authors read and approved the final manuscript.
